# The Immunoreactive Exo-1,3-β-Glucanase from the Pathogenic Oomycete *Pythium insidiosum* Is Temperature Regulated and Exhibits Glycoside Hydrolase Activity

**DOI:** 10.1371/journal.pone.0135239

**Published:** 2015-08-11

**Authors:** Angsana Keeratijarut, Tassanee Lohnoo, Thidarat Rujirawat, Wanta Yingyong, Thareerat Kalambaheti, Shannon Miller, Vipaporn Phuntumart, Theerapong Krajaejun

**Affiliations:** 1 Department of Pathology, Faculty of Medicine, Ramathibodi Hospital, Mahidol University, Bangkok, Thailand; 2 Research Center, Faculty of Medicine, Ramathibodi Hospital, Mahidol University, Bangkok, Thailand; 3 Molecular Medicine Program, Multidisciplinary Unit, Faculty of Science, Mahidol University, Bangkok, Thailand; 4 Department of Microbiology and Immunology, Faculty of Tropical Medicine, Mahidol University, Bangkok, Thailand; 5 Department of Biological Sciences, Bowling Green State University, Bowling Green, Ohio, United States of America; Friedrich Schiller University, GERMANY

## Abstract

The oomycete organism, *Pythium insidiosum*, is the etiologic agent of the life-threatening infectious disease called “pythiosis”. Diagnosis and treatment of pythiosis is difficult and challenging. Novel methods for early diagnosis and effective treatment are urgently needed. Recently, we reported a 74-kDa immunodominant protein of *P*. *insidiosum*, which could be a diagnostic target, vaccine candidate, and virulence factor. The protein was identified as a putative exo-1,3-ß-glucanase (Exo1). This study reports on genetic, immunological, and biochemical characteristics of Exo1. The full-length *exo1* coding sequence (2,229 bases) was cloned. Phylogenetic analysis showed that *exo1* is grouped with glucanase-encoding genes of other oomycetes, and is far different from glucanase-encoding genes of fungi. *exo1* was up-regulated upon exposure to body temperature, and its gene product is predicted to contain BglC and X8 domains, which are involved in carbohydrate transport, binding, and metabolism. Based on its sequence, Exo1 belongs to the Glycoside Hydrolase family 5 (GH5). Exo1, expressed in *E*. *coli*, exhibited ß-glucanase and cellulase activities. Exo1 is a major intracellular immunoreactive protein that can trigger host immune responses during infection. Since GH5 enzyme-encoding genes are not present in human genomes, Exo1 could be a useful target for drug and vaccine development against this pathogen.

## Introduction

The filamentous, aquatic, oomycetous microorganism, *Pythium insidiosum* [1–3], is unlike most other oomycetes, in that it infects humans and other animals, leading to a life-threatening disease, called pythiosis. The disease has been increasingly reported from tropical and subtropical areas around the world [2,3]. Clinicopathological features of pythiosis include arteritis of extremities (vascular pythiosis), keratitis (ocular pythiosis), and skin ulcer (cutaneous/subcutaneous pythiosis). Diagnosis of pythiosis is difficult. Antimicrobial chemotherapy is not effective against *P*. *insidiosum*. Immunotherapy using a *P*. *insidiosum* vaccine is available, but with limited efficacy [1,4,5]. Surgical removal of an infected organ (i.e., eye and leg) is the main treatment option [1]. Many patients die from uncontrolled and progressive infection. An effective treatment for patients with pythiosis is urgently needed. Better understanding the biology and pathogenesis of *P*. *insidiosum* could lead to better methods of infection control.

Many pathogens produce immunogenic proteins that are involved in important biological processes, host immunity, or pathogenesis. For example, *Cryptococcus neoformans* produces the immunogens MP84 and MP115 [6]. In the dimorphic fungus *Blastomyces dermatitidis*, the 120-kDa immunogen, BAD1, was identified as a surface protein that contributes to pathogenesis of blastomycosis [7]. The 65-kDa immunogen, Camp65p, from *Candida albicans* is a glucanase protein that functions as adhesin and virulence factor [8]. MP65 elicits protective immunity against *C*. *albicans*, and therefore, has been considered as a potential vaccine candidate [8,9].

A 74-kDa immunogen has been consistently identified in various strains of *P*. *insidiosum* [10]. The 74-kDa immunogen is recognized by sera from patients with pythiosis, but not sera from healthy individuals. By using proteomic and molecular genetic approaches, the 74-kDa immunogen was identified as a putative exo-1,3-β-glucanase (Exo1; formerly known as PinsEXO1 [11]), and a 924-bp partial Exo1-encoding sequence was cloned from genomic DNA (gDNA) of *P*. *insidiosum*, as reported by Krajaejun et al [12]. Details of the biological role of Exo1 in the life of *P*. *insidiosum* are unknown.

β-glucan is major cell wall component of fungi and oomycetes (including *P*. *insidiosum*) [13–16]. β-glucan-degrading enzymes, β-glucanases, can facilitate morphogenesis, cell wall remodeling, hyphal elongation, and growth, which are crucial for fungal physiology and virulence [8,13,14]. β-glucanase-encoding genes have been identified in oomycetes [16,17], but information on the structure and function of these genes is limited. Here, we aim to identify the full-length coding sequence of the *P*. *insidiosum*’s exo-1,3-beta glucanase gene (*exo1*; formerly known as *PinsEXO1* [11,18,19]), and immunologically and biochemically characterize its gene product. We found that *exo1* was up-regulated upon exposure to body temperature, and its gene product is a major intracellular immunoreactive protein that exhibited glycoside hydrolase activities. Detailed characterization of *exo1* could promote better understanding of *P*. *insidiosum*’s biology and pathogenesis.

## Materials and Methods

### Ethics statement

This study was approved by the Committee on Human Rights Related to Research Involving Human Subjects, at the Faculty of Medicine, Ramathibodi Hospital, Mahidol University (approval number MURA2006/418/NS_1:2_). An informed consent was not obtained from patients (from whom microorganisms and clinical specimens were obtained) because the data were analyzed anonymously, and the institutional ethics committee waived the need for written informed consent from the participants.

### Microorganisms and growth conditions

Seven strains of *P*. *insidiosum* (Pi-S, P06, P12, P16, P17, P24, and P29) were analyzed for this study. *P*. *insidiosum* was cultured on a Sabouraud dextrose (SD) agar supplemented with 20 mg/ml (1x) dextrose and incubated at two different temperatures (28 and 37°C) for 7 or 14 days. The organism was also cultured on a SD agar supplemented with diminished amount of dextrose [2 mg/ml (0.1x), and none (0x)] and incubated at 37°C for 7 days.

### Antigen preparation

Antigens were prepared using the method described by Krajaejun et al [10]. Briefly, *P*. *insidiosum* was cultured on SD agar for 7 days at 37°C. Several small agar pieces with actively-growing mycelium were transferred to 100 ml SD broth and shaken (150 rpm) at 37°C for 7 days. Merthiolate (0.02% wt/vol) was added to kill the organism before the hyphae were filtered through a Durapore membrane filter (0.22-μm pore size; Millipore). The resulting hyphae-free broth, containing PMSF (0.1 mg/ml) and EDTA (0.3 mg/ml), was called culture filtrate antigen (CFA). The separated hyphal mass was ground in a pre-cooled mortar in the presence of 25 ml of cold sterile distilled water containing PMSF (0.1 mg/ml) and EDTA (0.3 mg/ml). The hyphal lysate was centrifuged (4,800 x g) at 4°C, for 10 min. The supernatant, called soluble antigen from broken hyphae (SABH), was filtered through a Durapore membrane filter (0.22-μm pore; Millipore). SABH and CFA were concentrated ~80 fold using an Amicon Ultra-15 centrifugal filter (10,000 nominal-molecular-weight limit; Millipore). The concentrated CFA and SABH were stored at -80°C until use.

### Genomic DNA extraction

Seven-day-old *P*. *insidiosum* hyphae were harvested for gDNA extraction, using the conventional-extraction protocol described by Lohnoo et al [20]. Briefly, 500 mg of hyphal mat were ground in the presence of liquid nitrogen, and mixed with 10 ml of the extraction buffer [100 mM Tris-HCl (pH 8.0), 100 mM EDTA, 250 mM NaCl, 40 μg/ml proteinase K and 1% SDS]. The mixture was incubated at 55°C overnight. gDNA was extracted with phenol:chloroform:isoamyl alcohol (25:24:1), and then chloroform:isoamyl alcohol (96:4). gDNA was precipitated by isopropanol, washed with 70% ethanol, air dried, dissolved in 500 μl TE buffer, and treated with RNase. The resulting gDNA was extracted with chloroform:isoamyl alcohol (96:4), and precipitated with 3M sodium acetate and isopropanol. The pellet was washed with 70% ethanol, air dried, and dissolved in 50 μl TE buffer.

### RNA extraction

Total RNA of *P*. *insidiosum* was extracted, using the method described by Krajaejun et al [21] with some modifications. Briefly, hyphal mat, from each growth condition, was transferred to a 2-ml tube (100 mg hyphae/tube), snap-frozen in liquid nitrogen, and stored at -80°C until use. The frozen hyphae were disrupted with glass beads (~700–1,200 μm diameter, Sigma), using a Qiagen Tissue Lyzer MM301 (setting: 30 Hz for 2 min x 2 times). Total RNA was extracted using Trizol Reagents (Invitrogen), and purified using an RNeasy Mini kit (Qiagen) with on-column DNase digestion. The RNA purity and concentration were measured using a NanoDrop 2000 spectrophotometer (Thermo scientific), and the integrity was checked by agarose gel electrophoresis. The total RNA was kept at -80°C until use.

### Adaptor PCR

**Fig 1:**
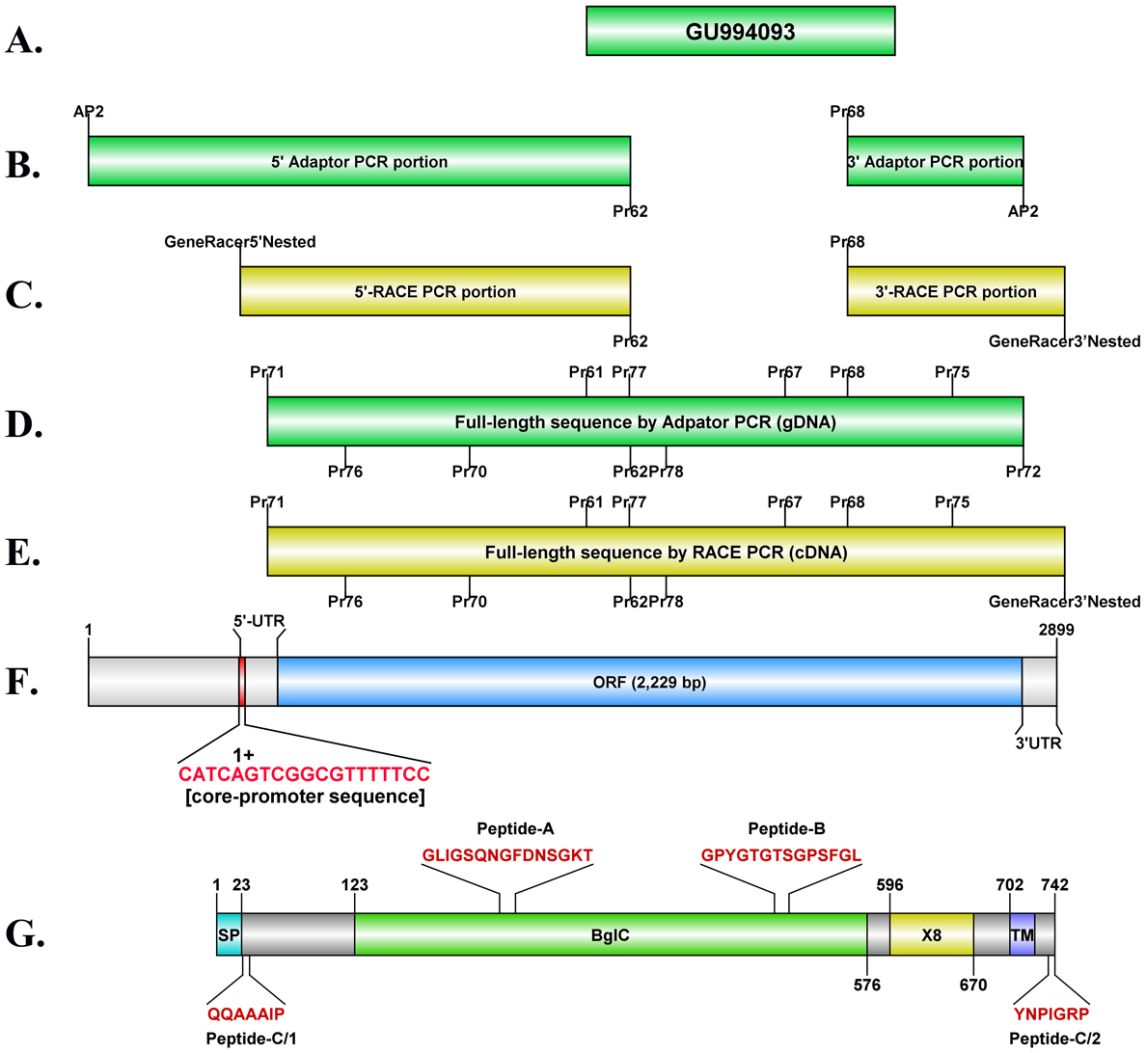
Identification and annotation of full length exo-1,3-β-glucanase gene (*exo1*) from *P*. *insidiosum*: (**A**) known 924-bp partial sequence of the *exo1* gene (accession number: GU994093); (**B**) Adaptor and (**C**) RACE PCR used to identify upstream and downstream regions of the partial *exo1* gene from genomic DNA (gDNA) and complementary DNA (cDNA), respectively; (**D)** and (**E**) Full length *exo1* coding sequence amplified from gDNA and cDNA, respectively [forward and reverse primers for PCR and sequencing (Table 1) are depicted above and below the sequence structure, respectively]; (**F**) Promoter [including the core-promoter sequence and the transcriptional start site (1+)], 5’-untranslated, open reading frame, and 3’-untranslated regions of the *exo1* gene; (**G**) Exo1 protein structure showing signal peptide (SP), BglC domain, X8 domain, transmembrane region (TM), and positions and sequences of Peptide-A, -B, and -C. Numbers indicate amino acid position.

Adaptor PCR was performed using the method described by Siebert et al [22]. Briefly, the adaptor-L sequence (5’-CTAATACGACTCACTATAGGGCTCGAGCGGCCGCCCGGGCAGGT-3’) and the adaptor-S sequence (5’-PO3-ACCTGCCC-NH2-3’) were mixed to final concentration of 100 pmol/μl, heated to 95°C for 5 min, and then cooled down to room temperature to generate the double-stranded flanking-sequence adaptor. *P*. *insidiosum*’s gDNA was digested by each of these blunted-end restriction enzymes (20 unit/reaction): *Dra*I, *Eco*RV, *Pvu*II, *Sca*I, *Sma*I, *Nru*I, *Stu*I, *Hpa*I, and *Nae*I (New England Biolabs). Digested gDNAs (~1 μg) were ligated with the flanking-sequence adaptor (100 pmol), using T4 DNA ligase (Promega). First-round PCR amplifications (25-μL reaction) contained 50 ng adaptor-ligated gDNA, 0.4 mM dNTPs (Promega), 0.2 mM each of the outer adaptor primer AP1 and a first-round *exo1*-specific primer (Pr78 for 5’ fragment; Pr67 for 3’ fragment) (Table 1; Fig 1B and 1D), and 0.5 μL of Elongase DNA polymerase (Invitrogen) in 1x Elongase buffer (buffer A:B = 1:4). The reaction was carried out in a MyCycler thermal cycler (Biorad) with the following setting: 94°C for 1 min, 35 cycles of 94°C for 30 s, 60°C for 2 min, and 68°C for 4 min, and then 68°C for 15 min. The PCR products were assessed by 1% agarose gel electrophoresis. If more than one PCR product were observed, a second-round PCR amplification (25-μL reaction) was then performed using 1 μl of 1/100 dilution of primary PCR product, 0.4 mM dNTPs (Promega), 0.2 mM each of the inner adaptor primer AP2 and a second-round *exo1*-specific primer (Pr62 for 5’ fragment; Pr68 for 3’ fragment) (Table 1; Fig 1B and 1D), 0.5 μL of Elongase DNA polymerase (Invitrogen) in 1x Elongase buffer (buffer A:B = 1:4), and the following condition: 94°C for 1 min, 20–25 cycles of 94°C for 30 s, 60°C for 2 min, and 68°C for 4 min, and then 68°C for 15 minutes.

**Table 1:** List of primers used in this study.

Primer	Sequence
GeneRacer 5’	5’-CGACTGGAGCACGAGGACACTGA-3
GeneRacer 5’ Nested	5’-GGACACTGACATGGACTGAAGGAGTA-3′
GeneRacer 3’	5’-GCTGTCAACGATACGCTACGTAACG-3′
GeneRacer 3’ Nested	5’-CGCTACGTAACGGCATGACAGTG-3’
AP1	5’-GGATCCTAATACGACTCACTATAGGGC-3’
AP2	5’-AATACGACTCACTATAGGGCTCGAGAGGC-3’
Pr61	5’-AACTACGGCAACCTGAAC-3’
Pr62	5’-TCTTGAGCACCTTGATGG-3’
Pr67	5’-TTTGCCCCGTGAACAGCAACCTC-3’
Pr68	5’-GCCCAAGAACATCTCGGACTATG-3’
Pr70	5’-GTCCTCCGTGACCCAGTTCGC-3’
Pr71	5’-CACGTGAGCCCGTCCTGTGATC-3’
Pr72	5’-GTGCCTATGGGCGGCCGATGG-3’
Pr75	5’-TCAAGCCCTCGCAGATCAACAC-3’
Pr76	5’-CTTGGGCTCGGCCTTGGCTT-3’
Pr77	5’-AAGACGTACTACTGGAAG-3’
Pr78	5’-CATAAAGTCGAGCCAGAA-3’
Pr79	5’-GACCTCCCCTTATCAATCATGGC-3’
Pr80	5’-CTTGCACATGCCGGAGCC-3’
PinsEXO1BamHI	5’-CAGCAGGGATCCCTGATCCCTCGCCCAC-3’
PinsEXO1NcoI	5’-GCGGCCCCATGGGTTGTACTGCTGGCGA-3’

### RACE PCR

cDNAs were generated from *P*. *insidiosum*’s RNA using a GeneRacer kit (Invitrogen). Briefly, 4 μg of total RNA underwent dephosphorylation using calf intestinal phosphase, removal of the mRNA cap using tobacco acid pyrophosphatase, ligation of the GeneRacer RNA oligo (5′-CGACUGGAGCACGAGGACACUGACAUGGACUGAAGGAGUAGAAA-3′) using T4 RNA ligase, and cDNA synthesis using the SuperScript III reverse transcriptase and the GeneRacer Oligo dT primer (Table 1; Fig 1C and 1E). The resulting cDNAs were used as templates for 25-μL-reaction RACE PCR, comprising 0.4 mM dNTPs (Promega), 0.5 μL Elongase DNA polymerase (Invitrogen) in 1x Elongase buffer (buffer A:B = 1:4), 0.2 μM each of the primer GeneRacer 5’ and the *exo1*-specific primer Pr78 (or the primer GeneRacer 3’ and the *exo1*-specific primer Pr67) (Table 1; Fig 1C and 1E) (the Biorad MyCycler thermal cycler setting: 94°C for 2 min, 35 cycles of 94°C for 15 s, 55–60°C for 1 min, and 68°C for 2.5 min, and then 68°C for 10 min). PCR products were assessed by 1% agarose gel electrophoresis. If more than one PCR product were observed, a second-round PCR amplifications (25-μL reaction) was then performed as mentioned above, using 1 μl of primary PCR product, 0.2 μM each of the primer GeneRacer 5’ Nested and Pr62 (or the primer GeneRacer 3’ Nested and Pr68).

### Amplification and sequencing of full-length *exo1*

Full-length *exo1* gene sequence was PCR amplified from gDNA of *P*. *insidiosum* (strain P06, P16, P17, P24, P29, and Pi-S) using the primer Pr71 and Pr72, and from cDNA of *P*. *insidiosum* (strain P06) using the primer Pr71 and GeneRacer 3’ Nested (Table 1; Fig 1D and 1E). PCR amplification was performed in a 25-μL reaction, comprising 100 ng template, 0.4 mM dNTPs (Promega), 0.5 μL Elongase DNA polymerase (Invitrogen) in 1x Elongase buffer (buffer A:B = 1:4), 0.2 μM each of the primers. The reaction was carried out in a Biorad MyCycler thermal cycler, using the following condition: 94°C for 2 min, 30 cycles of 94°C for 30 s, 55°C (gDNA template) or 58.5°C (cDNA template) for 2 min, and 68°C for 3 min, and then 68°C for 15 min. PCR product was analyzed by 1% agarose gel electrophoresis. Direct sequencing of the full-length *exo1* was performed using the Big Dye Terminator v. 3.1 Cycle Sequencing Kit (Applied Biosystems). Primers AP2, GeneRacer 5’ Nested, GeneRacer 3’ Nested, Pr61, Pr62, Pr67, Pr68, Pr70, Pr71, Pr72, Pr75, Pr76, Pr77, and Pr78, were used to obtain the full-length coding sequence of *exo1* (Table 1; Fig 1). Automated sequencing was performed using an ABI 3100 Genetic Analyzer (Applied Biosystems) and analyzed using the Applied Biosystems Sequencing software.

### Bioinformatics and phylogenetic analyses

The *exo1* open reading frame (ORF) was determined and translated to protein, using the ORF finder program (http://www.ncbi.nlm.nih.gov/projects/gorf/). Molecular weight of the Exo1 protein was calculated online at http://www.bioinformatics.org/sms/prot_mw.html. Signal peptide and transmembrane domain were predicted using the SignalP program version 4.1 (http://www.cbs.dtu.dk/services/SignalP/) and the TMHMM program version 2.0 (http://www.cbs.dtu.dk/services/TMHMM/). The NCBI’s conserved domain database [23] and the InterProScan [24] were employed to search protein domains of Exo1. The DOG program version 2.0 was used to draw gene and protein structures [25]. Glucanase gene and peptide sequences were BLAST searched against the *P*. *insidiosum* transcriptome [21] or draft genome [26].

Six *exo1* sequences from *P*. *insidiosum* strains (Pi-S, P06, P16, P17, P24, and P29), and 35 top *exo1*-BLAST hit DNA sequences from 9 oomycetes and 26 fungi [27] (Table 2), were aligned for phylogenetic analysis, using the MUSCLE program (v3.7) [28]. Ambiguous regions (i.e., gaps or poor alignment) were removed using the Gblocks program (v0.91b) [29]. The phylogenetic reconstruction was performed using the PhyML program (v3.0) [30]. Internal branch reliability was determined using the aLRT test (SH-Like) [31]. The tree was visualized using the TreeDyn program (v198.3) [32]. All the programs were executed online at www.phylogeny.fr [33].

**Table 2:** List of oomycete and fungal microorganisms whose top BLAST-hit DNA sequences vs. the *P*. *insidiosum*’s *exo1* gene were used for phylogenetic analysis. All sequences were retrieved from the FungiDB database [27], and BLAST searched against the NCBI nucleotide database.

Microorganism	Group	FungiDB gene ID	BLAST search against NCBI database			
			Description	Accession	*E*-value	Identity (%)
*Pythium insidiosum* P06	Oomycetes	-	exo-1,3-beta-glucanase	LC033486	0	78%
*Pythium insidiosum* P16	Oomycetes	-	exo-1,3-beta-glucanase	LC033488	0	78%
*Pythium insidiosum* P17	Oomycetes	-	exo-1,3-beta-glucanase	LC033491	0	78%
*Pythium insidiosum* P24	Oomycetes	-	exo-1,3-beta-glucanase	LC033489	0	78%
*Pythium insidiosum* P29	Oomycetes	-	exo-1,3-beta-glucanase	LC033490	0	78%
*Pythium insidiosum* Pi-S	Oomycetes	-	exo-1,3-beta-glucanase	LC033487	0	78%
*Pythium ultimum*	Oomycetes	PYU1_G011796	exo-1,3-beta-glucanase	XM_009541535.1	0	77%
*Saprolegnia parasitica*	Oomycetes	SPRG_13455	exo-1,3-beta-glucanase	XM_009541535.1	0	75%
*Hyaloperonospora arabidopsidis*	Oomycetes	HpaG806582	exo-1,3-beta-glucanase	AF494014.1	0	81%
*Phytophthora capsici*	Oomycetes	PHYCA_532433	exo-1,3-beta-glucanase	AF494014.1	0	83%
*Phytophthora cinnamomi*	Oomycetes	PHYCI_330310	exo-1,3-beta-glucanase	XM_009533109.1	0	90%
*Phytophthora infestans*	Oomycetes	PITG_09798	glucan 1,3-beta-glucosidase	XM_002903345.1	0	100%
*Phytophthora parasitica*	Oomycetes	PPTG_01939	exo-1,3-beta-glucanase	AF494014.1	0	90%
*Phytophthora ramorum*	Oomycetes	PSURA_41489	exo-1,3-beta-glucanase	XM_009533109.1	0	86%
*Phytophthora sojae*	Oomycetes	PHYSO_564063	exo-1,3-beta-glucanase	XM_009533109.1	0	100%
*Ajellomyces capsulatus*	Fungi	HCBG_00519	glucan 1,3-beta-glucosidase	XM_001540694.1	0	97%
*Aspergillus fumigatus*	Fungi	Afu1g03600	exo-beta-1,3-glucanase	XM_745017.1	0	100%
*Botryotinia fuckeliana*	Fungi	BC1G_01483	glucan 1,3-beta-glucosidase	XM_001593065.1	0	87%
*Candida albicans*	Fungi	orf19.2990	exo-1,3-beta-glucanase	X56556.1	0	100%
*Candida glabrata*	Fungi	CAGL0G09515g	exo-1,3-beta-glucanase	FR847041.1	1.00E-162	72%
*Coccidioides immitis*	Fungi	CIHG_01427	beta-glucosidase 6	XM_003065912.1	0	99%
*Coprinopsis cinerea*	Fungi	CC1G_06563	exo-beta-1,3-glucanase	XM_001829174.1	0	100%
*Cryptococcus neoformans*	Fungi	CNE03150	exo-beta-1,3-glucanase	XM_001829174.1	4.00E-29	69%
*Fusarium graminearum*	Fungi	FGSG_08623	exo-beta-1,3-glucanase	XM_007811660.1	0	72%
*Malassezia globosa*	Fungi	MGL_3222	exo-beta-1,3-glucanase	XM_001829175.1	2.00E-13	70%
*Melampsora larici-populina*	Fungi	MELLADRAFT_47334	exo-1,3-beta-glucanase	XM_001385723.1	9.00E-18	74%
*Mucor circinelloides*	Fungi	QYA_128967	exo-beta—glucanase	XM_007852469.1	3.00E-05	78%
*Neosartorya fischeri*	Fungi	NFIA_021060	exo-beta-1,3-glucanase	XM_001265294.1	0	100%
*Neurospora crassa*	Fungi	NCU03914	glucan 1,3-beta-glucosidase	XM_009650465.1	0	72%
*Phanerochaete chrysosporium*	Fungi	PHCHR_130958	exo-beta-1,3-glucanase	XM_007360605.1	0	76%
*Phycomyces blakesleeanus*	Fungi	PHYBL_155691	exo-beta-1,3-glucanase	XM_003856315.1	2.00E-20	72%
*Puccinia graminis*	Fungi	PGTG_04377	exo-1,3-beta-glucanase	XM_002491316.1	6.00E-20	73%
*Saccharomyces cerevisiae*	Fungi	YOR190W	exo-1,3-beta-glucanase	S52935.1	0	100%
*Sclerotinia sclerotiorum*	Fungi	SS1G_06037	glucan 1,3-beta-glucosidase	XM_001593065.1	0	100%
*Sordaria macrospora*	Fungi	TRIREDRAFT_64375	exo-beta-1,3-glucanase	XM_007811660.1	0	73%
*Sporisorium reilianum*	Fungi	r11587	exo-beta-1,3-glucanase	XM_007360605.1	5.00E-40	70%
*Talaromyces marneffei*	Fungi	PMAA_055430	exo-beta-1,3-glucanase	XM_002150320.1	0	100%
*Talaromyces stipitatus*	Fungi	TSTA_017460	exo-beta-1,3-glucanase	XM_002483860.1	0	100%
*Tremella mesenterica*	Fungi	TREME_32166	exo-beta-1,3-glucanase	XM_007269119.1	8.00E-25	68%
*Ustilago maydis*	Fungi	um00235	exo-beta-1,3-glucanase	XM_001829174.1	1.00E-34	70%
*Yarrowia lipolytica*	Fungi	YALI0_F05390g	exo-1,3-beta-glucanase	Z46872.1	0	99%

### Measurement of *exo1* transcript

cDNA was synthesized, using a reverse transcription system kit (Promega), in a 20-μl reaction, comprising 1,000 ng total RNA, 1 μl random hexamer, 1 mM dNTPs, 0.5 μl recombinant RNasin ribonuclease inhibitor, 0.5 μl AMV reverse transcriptase, 1x reverse transcription buffer, and nuclease-free water. The reaction was performed in a Biorad MyCycler thermal cycler, using the following conditions: 25°C for 10 min, 42°C for 30 min, 95°C for 5 min. The primer Pr77 and Pr78 were used to amplify *exo1*, while the primer Pr79 and Pr80 were used to amplify the *P*. *insidiosum*’s actin gene, *act1* (accession number: HS975373). Real-time PCR was performed in a 20-μl reaction, comprising 100 ng cDNA template, 10 mM each primer, 1x SsoFast EvaGreen (Biorad), and RNase-free water, using a CFX96 Touch real-time PCR machine (BioRad) with the following conditions: the initial denaturation at 95°C for 3 min, 40 cycles of 95°C for 10 s and 57°C for 30 s, the final extension at 65°C for 5 s, and then 95°C for 5 s. A reaction without cDNA (no-template control) served as negative control. Each reaction was performed in triplicate. Expression of *exo1*, in relation to *act1*, was analyzed using the CFX 3.0 relative normalized expression program (Biorad).

### Synthetic peptides and rabbit anti-Exo1 peptide serum

B-cell epitopes of the Exo1 protein were predicted using the PREDITOP program [34]. Three predicted peptides (Peptide-A, GLIGSQNGFDNSGKT; Peptide-B, GPYGTGTSGPSFGL; and Peptide-C, QQAAAIPCYNPIGRP) were synthesized (>95% purity) and conjugated with keyhole limpet hemocyanin (Mimotopes, Australia). Rabbit antisera, raised against a pool of these three peptides (anti-Exo1 peptide serum), was purchased from Mimotopes. ELISA titer of the rabbit antisera against each peptide was measured, using the Mimotopes’s protocol.

### ELISA

Serum samples from three pythiosis patients (who were diagnosed by culture identification or serological tests [35]) and three healthy blood donors (who came to the Blood Bank Division, Ramathibodi Hospital) were used for ELISA analyses. Wells of 96-well polystyrene plate were coated with 100 μl/well of 5 μg/ml of either Peptide-A, -B or -C, or with a pool of these peptides (1:1:1) in coating buffer (0.1 M carbonate buffer, pH 9.6), and incubated overnight at 4°C. After washing the plate four times with PBS-T buffer [phosphate buffered saline (pH 7.4), 0.5% Tween20], 100 μl of blocking buffer (1% casein in phosphate buffer solution, pH 9.6) was added to each well, incubated at 37°C for 1 hr, and washed four times with PBS-T. Serum sample (1:1,600 in 1% casein buffer) was added to each well (100 μl/well), incubated at 37°C for 2 hr, and washed four times with PBS-T. Each serum sample was tested in duplicate. Goat anti-human immunoglobulin G conjugated with horseradish peroxidase (Biorad; 1:100,000 in 1% casein buffer) was added (100 μl/well), and incubated at 37°C for 2 hr. After washing the plate four times with PBS-T, chromogen [20 μl of 0.6% TMB and 1 ml of 0.009% H_2_O_2_ in acetate buffer] was added at 100 μl/well, and incubated at room temperature for ~5 min. The reaction was terminated by adding 100 μl/well of 0.3 N sulfuric acid. Optical density was measured using an Infinite200 Pro ELISA reader (Tecan, Austria) at the wavelength of 595 nm. The cutoff value for a positive signal was calculated from the mean of the control ELISA signals plus 4 SDs, as reported by Chareonsirisuthigul et al [35].

### SDS-PAGE and Western blot

Proteins of SABH, CFA and cell lysate of Exo1-expressing *E*. *coli* (10 μg/lane) were separated by SDS-PAGE (4% stacking gel and 12% resolving gel), using a Biorad MiniProteon II apparatus (setting: 100 V, for 90 min) and blotted onto a polyvinylidene difluoride (PVDF) membrane (0.2-μm pore size; Millipore), using a Biorad Mini Trans-Blot cell apparatus (setting: 100 V for 60 min). The PVDF membrane was blocked with 5% nonfat-dried milk in TBS-T buffer (1.0 M Tris-base, 1.5 M NaCl, and 1.0% Tween 20) for 1 hr at room temperature, and washed once with TBS-T. The membrane was incubated with rabbit anti-Exo1 peptide serum (1:2,000 in 5% nonfat-dried milk in TBS-T) at 37°C for 1 hr. After the membrane was washed twice with TBS-T, goat anti-rabbit IgG conjugated with alkaline phosphatase (Southern Biotech; 1:4,000 in 5% nonfat-dried milk in TBS-T) was added to the membrane, and incubated at 37°C for 60 min. The membrane was washed three times with TBS-T. Western blot signals were developed using NBT and BCIP (Roche).

To block the anti-Exo1 antibodies in the rabbit immune serum, 3 ml of diluted rabbit serum (1:1,000 in 1% BSA in TBS-T) and a combination of Peptide-A, -B, or -C (5 μg each), were incubated at 4°C overnight, and then centrifuged at 440 x g. The resulting pre-absorbed serum was used in Western blot, as mentioned above.

### *exo1* expression in *E*. *coli*

*exo1* coding sequence was amplified from the *P*. *insidiosum* strain P06 (CBS119452) in a 25-μl PCR reaction, containing 100 ng gDNA, 0.5 μl Elongase (Invitrogen) and its 1x buffer (buffer A:B = 1:4), 0.4 μM dNTPs, and 0.2 μM each of the primer PinsEXO1BamHI and PinsEXO1NcoI (Table 1). The reaction was carried out in a MyCycler thermal cycler (Biorad), using the following conditions: 94°C for 1 min, 35 cycles of 94°C for 30 s, 55°C for 2 min, and 68°C for 4 min, and then 68°C for 15 min. The PCR product was purified using a PCR cleanup kit (GeneAid), double digested with *Bam*HI and *Nco*I (New England Biolabs), and directionally cloned into pRSET-C (Invitrogen). The resulting plasmid, pPinsEXO1, was checked for correct in-frame translation, and transformed into the *Escherichia coli* strain BL21 (DE3) pLys for protein expression.

A bacterial clone, containing pPinsEXO1, was grown in LB broth supplemented with ampicillin (50 μg/ml). The bacteria were incubated with shaking (200 rpm) at 37°C for ~3 hr to an optical density (600 nm) of 0.6–0.8. Protein expression was induced with 1 mM IPTG (Invitrogen) at 37°C for another 3 hr. Bacteria were harvested by centrifugation (6,000 x g) at 4°C for 15 min, and disrupted with BugBuster protein extraction reagent (Novagen) and sonication. The cell lysate was centrifuged and the induced protein was enriched from the supernatant using a Ni-NTA agarose column (Qiagen) and elution with immidazole. The eluted proteins underwent SDS-PAGE and Western blot analyses, using 1:5,000 mouse anti-6x histidine (anti-6xHis) antibody (Southern Biotech) and 1:4,000 goat anti-mouse IgG conjugated with alkaline phosphatase (Southern Biotech).

### Agar plate enzymatic assay

The *E*. *coli* strain BL21 (DE3) pLyS, containing pPinsEXO1 or pRSET-C empty plasmid, were grown in LB broth supplemented with ampicillin (50 μg/ml). IPTG (final concentration, 1 mM) was added to the culture to induce protein expression. After incubation at 37°C for 4 hr, several cell suspensions (bacteria with pPinsEXO1) were prepared, ranging from 1 x 10^2^ to 1 x 10^9^ cells/ml (1 OD_600_ = 10^9^ cells/ml), and 100 μl of each cell suspension were spotted onto a 6-mm-diameter antibiotic assay disc (Whatman) placed on a LB agar mixed with 1.5% (w/v) of Avicel PH-101 (Fluka-Sigma), or a LB agar overlaid with 100 μl of 0.5% (w/v) Laminarin (Sigma). Positive controls included either 100 μl (concentration: 1 x 10^2^–1 x 10^−9^ mg/ml) of *Trichoderma reesei*’s cellulase (Sigma) or *Trichoderma harzianum*’s lysing enzyme (containing β-glucanase, cellulase, protease, and chitinase activities; Sigma). Negative controls included 100 μl of plain LB broth, or bacteria with pRSET-C (1 x 10^9^ cells/ml). After incubation at 37°C overnight, LB agar was stained with iodine-staining solution [2 g potassium iodide (Carlo Erba) and 1 g iodine (BDH Laboratory) in 300 ml of distilled water] [36]. Diameter of the hydrolytic (clear) zone generated by each sample was measured. All samples were tested in triplicate.

### Nucleotide sequence accession numbers

All *exo1*-coding sequences from *P*. *insidiosum* strain P06, Pi-S, P16, P24, P29, and P17 have been submitted to the DNA Data Bank of Japan database, under accession numbers LC033486 to LC033491, respectively.

## Results

### Structure of *exo1* and its gene product

Upstream (1,491-bp long) and downstream (484-bp long) regions of the partial *exo1* gene sequence (924-bp long; accession number, GU994093; Fig 1A) were obtained using adaptor PCR (for gDNA; Fig 1B and 1D) and RACE PCR (for cDNA; Fig 1C and 1E) techniques. Alignment of the gDNA- and cDNA-derived sequences demonstrated that *exo1* has no introns, and comprises 112-bp-long 5’-untranslated region, 2,229-bp-long ORF, and 107-bp-long 3’-untranslated region (Fig 1F). BLAST searches of the *exo1* ORF against a draft *P*. *insidiosum* genome database [26] identified sequences in the contig#0076 (bit score, 3896; E-value, 0.0; identity, 99%) and the contig#0165 (bit score, 3896; E-value, 0.0; identity, 98%). The upstream region (between 99 and 117 nucleotides) of the *exo1* start codon includes a short sequence (5’-CATCAGTCGGCGTTTTTCC-3’; Fig 1F) that is similar to the 19-nucleotide oomycete core-promoter sequence (5’-GCYCATTYYNCAWTTTNYY-3’) [37], as indicated by underlines. This short sequence contains two possible core-promoter components: an initiator element (5’-TCAGTCG-3’; the adenosine triphosphate (1+) is a predicted transcription start site; Fig 1F) and a flanking promoter region (5’-CGTTTTTCC-3’).

The *exo1* open reading frame encodes a 742-amino-acid protein (Fig 1G). The SignalP program indicated that the first 23 N-terminus amino acids of the Exo1 protein is a signal peptide. Calculated molecular weights of Exo1, with and without the signal peptide, are 82.2 kDa and 79.8 kDa, respectively. Exo1 contains BglC domain (amino acid positons: 123–576; domain description: endoglucanase [carbohydrate transport and metabolism]; its catalytic domain belongs to the glycoside hydrolase family 5 [GH5]), and X8 domain (amino acid positons: 596–670; domain description: possible involvement in carbohydrate binding). The amino acid positon 702–724 are predicted to be a transmembrane region.

**Fig 2:**
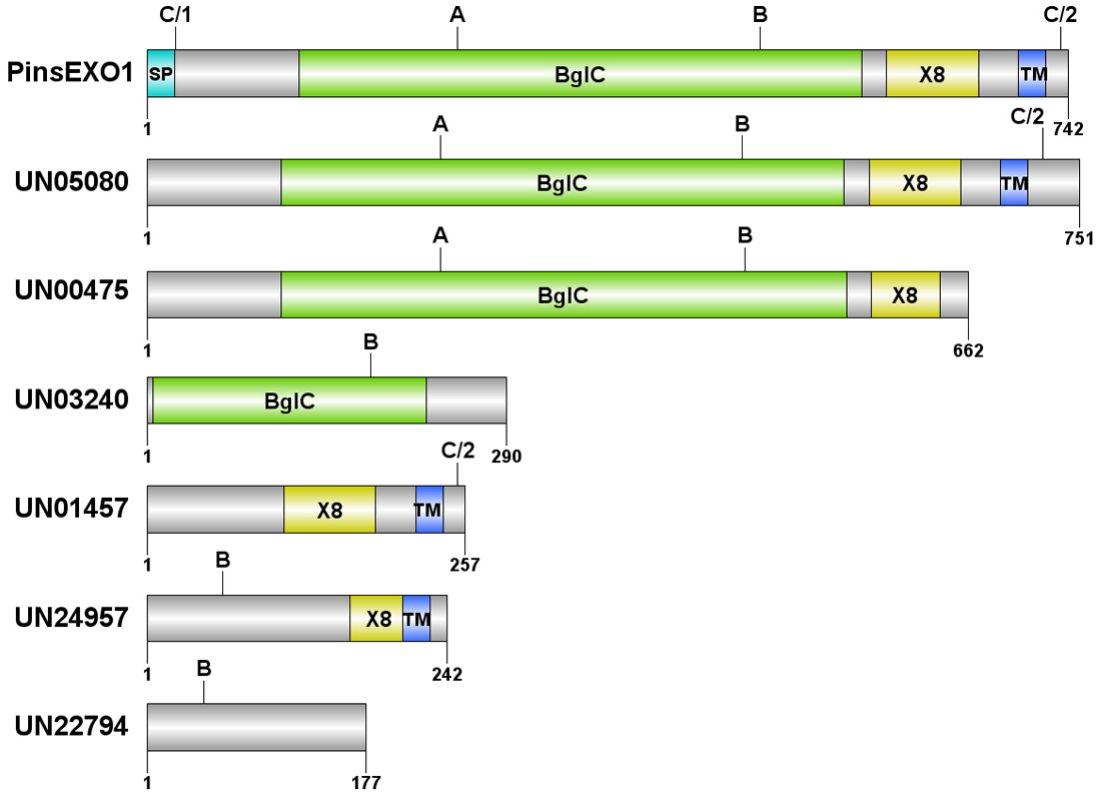
Protein structure of Exo1 and six transcriptome-derived homologous proteins. Protein domains of Exo1 and homologous proteins (UN05080, UN00475, UN03240, UN01457, UN24957 and UN22794; Table 3) were predicted by SignalP, TMHMM, and NCBI’s conserved domain search programs (Methods). The DOG program was used to draw protein structures. Numbers indicate the first and last amino acid positions of each protein. Detailed characteristics of the homologous proteins are shown in Table 3. (Symbols: A, Peptide-A; B, Peptide-B; C/1, Peptide-C (the first half portion); C/2, Peptide-C (the second half portion); SP, signal peptide; BglC, BglC domain; X8, X8 domain; and TM, transmembrane region; The grey regions are sequences that do not match any protein domain defined by the NCBI’s conserved domain search program).

The PREDITOP program [34] predicts Peptide-A and -B (Fig 1G) to be B-cell epitopes. Peptide-C is a chimeric peptide comprising two short amino acid regions from N-terminus end (QQAAAIP) and C-terminus end (YNPIGRP) of Exo1 (Fig 1G). BLAST search of these peptides against the *P*. *insidiosum* transcriptome [21] indicated that the peptides have homologies to several different transcripts representing different glucanase genes of *P*. *insidiosum*. All three peptides had matches to the transcript #UN05080; whereas only Peptide-A and Peptide-B had matches in the transcript #UN00475; Peptide-B alone was found in transcripts #UN03240, UN24957 and UN22794; and finally, only Peptide-C was found in transcript #UN01457 (Table 3). NCBI accession number, predicted protein length, calculated protein molecular weight, number of 454-derived transcript reads (when *P*. *insidiosum* grew at 37°C [21]), and sequence alignment analysis against Exo1 (as the query sequence), corresponding to each transcriptome-derived protein (as the subject sequence), are summarized in Table 3. Predicted protein structure (size and domain) of Exo1 and the transcriptome-derived proteins are shown in Fig 2.

**Table 3:** *P*. *insidiosum*’s transcriptome-derived Exo1 homologous proteins that share the Peptide-A, -B, or -C sequences. Predicted structures of these proteins are shown in Fig 2. NCBI accession number, protein length, calculated protein molecular weight (MW), number of 454-derived transcript reads (when *P*. *insidiosum* grew at 37°C), and sequence alignment analysis against Exo1 (including: query sequence coverage, *E*-value, and sequence identity), corresponding to each transcriptome-derived protein, are summarized in the table.

Subject sequence	Accession number	Protein length (amino acids)	MW (kDa)	Number of transcript reads	Exo1 peptide			Alignment against Exo1 (Query sequence)		
					A	B	C	Query coverage ^a^	*E*-value	Sequence identity ^b^
UN05080	FX532070	751	82.8	109	Yes	Yes	Yes	98%	0.0	93%
UN00475	FX527465	682	76.1	17	Yes	Yes	-	86%	0.0	92%
UN03240	FX530230	290	32.7	1	-	Yes	-	38%	0.0	97%
UN01457	FX528447	257	28.3	1	-	-	Yes	37%	5.00E-174	93%
UN24957	FX551947	242	26.9	4	-	Yes	-	32%	2.00E-157	100%
UN22794	FX549784	177	19.6	1	-	Yes	-	20%	3.00E-87	88%

^a^ Percent length value of a query sequence (i.e., Exo1) that covers or can align with a subject sequence

^b^ Percent identity value of query (Exo1) and subject sequences within the Query coverage region

**Fig 3:**
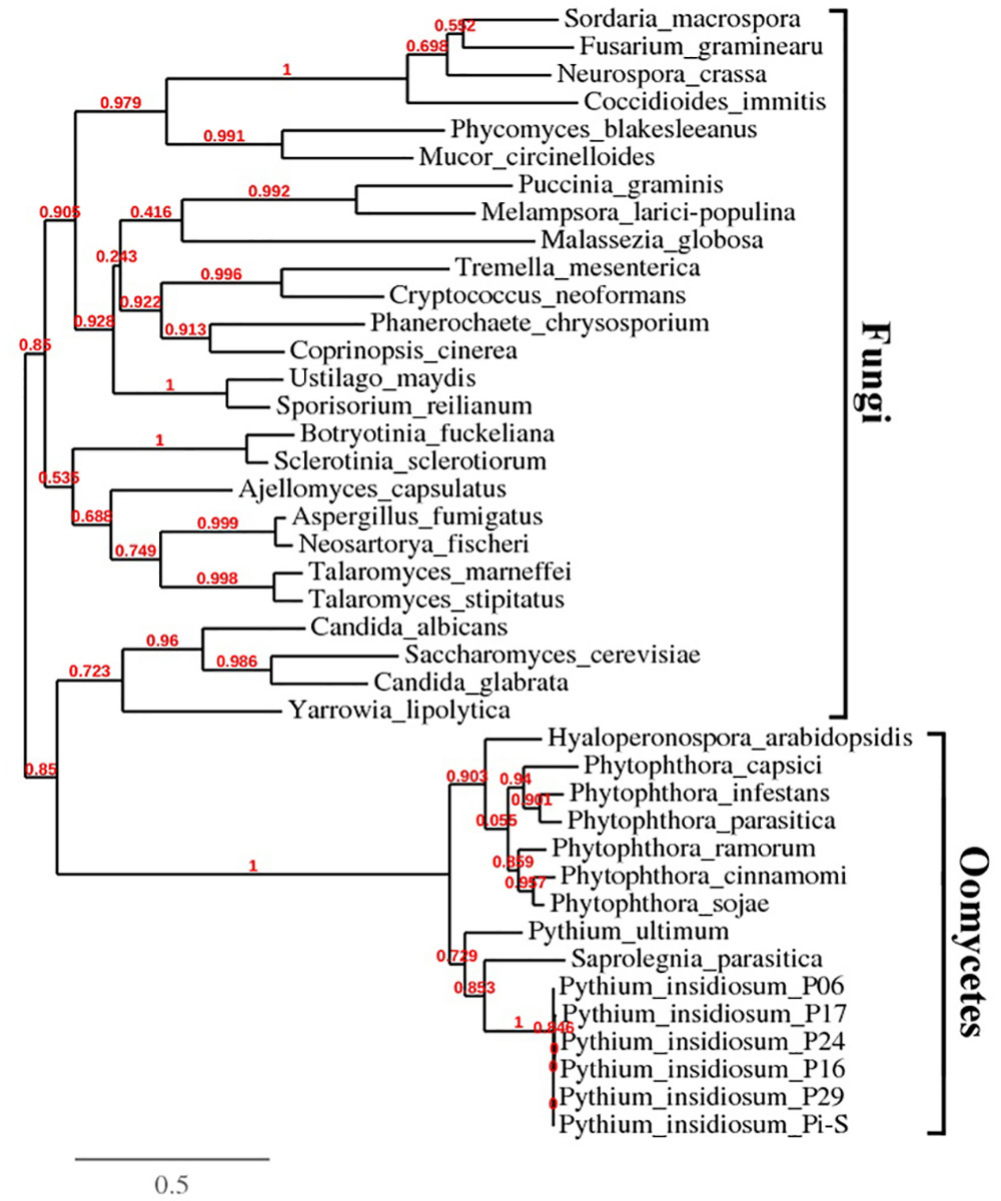
Phylogenetic analysis of glucanase genes from oomycetes and fungi. *exo1* gene sequences from 6 strains of *P*. *insidiosum* (accession number: LC033486 to LC033491), and glucanase-encoding genes (top *exo1*-BLAST hit sequences) from 9 other oomycetes and 26 fungi (Table 2) were included for phylogenetic analysis. Phylogenetic reconstruction was performed using the PhyML program (Methods). Reliability for internal branch was analyzed using the aLRT test (Methods).

The *exo1*-coding sequences (accession number: LC033486 to LC033491) from six strains of *P*. *insidiosum*, and 35 top *exo1*-BLAST hit sequences from 9 oomycetes and 26 fungi [27] (Table 2), were included for phylogenetic analysis. In the resulting tree, the sequences from *P*. *insidiosum* and other oomycetes form a clade that is separate from the sequences of fungi (Fig 3). Among the oomycetes, all *exo1* sequences group together, and their phylogenetic positions placed them closer to the sequences from *S*. *parasitica* and *P*. *ultimum* than the sequences from other oomycetes.

### Impact of temperature, culture duration, and dextrose availability on *exo1* expression

**Fig 4:**
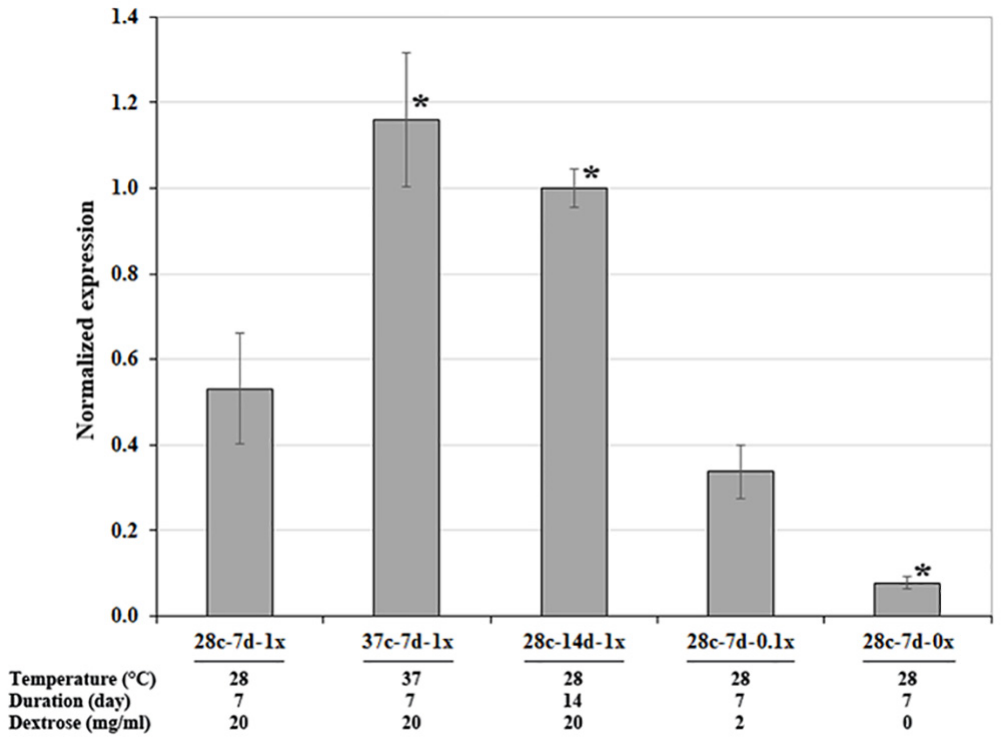
Expression of *exo1* in response to temperature, culture duration, and dextrose availability. Real-time PCR was used to measure *exo1* mRNA levels in *P*. *insidiosum* (strain Pi-S) at 5 culture conditions: (i) 28°C for 7 days (28c-7d-1x); (ii) 37°C for 7 days (37c-7d-1x); (iii) 28°C for 14 days (28c-14d-1x); and 28°C for 7 days with (iv) 2 mg/ml dextrose (28c-7d-0.1x), or (v) no dextrose (28c-7d-0x) supplement. *exo1* expression in each condition was normalized to the reference actin gene (*act1*). Asterisk indicates significant up- or down-regulation, relative to 28c-7d-1x.

The *P*. *insidiosum* strain Pi-S was incubated in SD broth under 5 different combinations of temperature, culture durations, and dextrose supplementation. Total RNAs extracted from *P*. *insidiosum*, grown in each condition, were converted to cDNAs. Real-time PCR was used to determined expression of *exo1*, relative to expression of the actin-encoding reference gene, *act1*. Compared to the condition 28c-7d-1x, normalized expression of *exo1* was significantly increased in 37c-7d-1x (2.2-fold higher) and 28c-14d-1x (1.9-fold higher), while the normalized expressions of *exo1* in 28c-7d-0x was significantly decreased (0.9-fold lower) (Fig 4). Similar results were observed with the *P*. *insidiosum* strain P06 (data not shown). These findings indicate that *exo1* transcript accumulates over culture time and is regulated by temperature and carbon source availability.

### Exo1 is an intracellular immunogen

**Fig 5:**
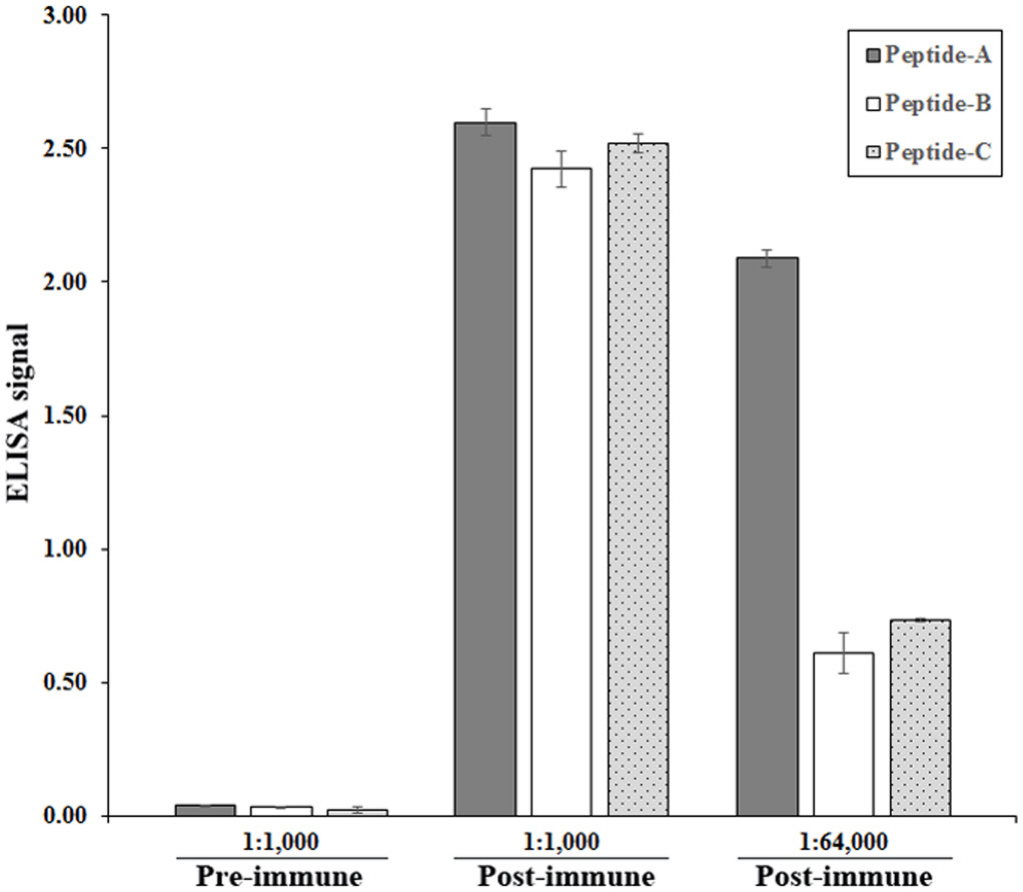
Immunoreactivity of Exo1 peptides against rabbit anti-Exo1 peptide sera. ELISA result of rabbit pre-immune or anti-Exo1 peptide serum (raised against the combination of Peptide-A, -B, and -C) with the individual peptides (used to coat an ELISA plate).

**Fig 6:**
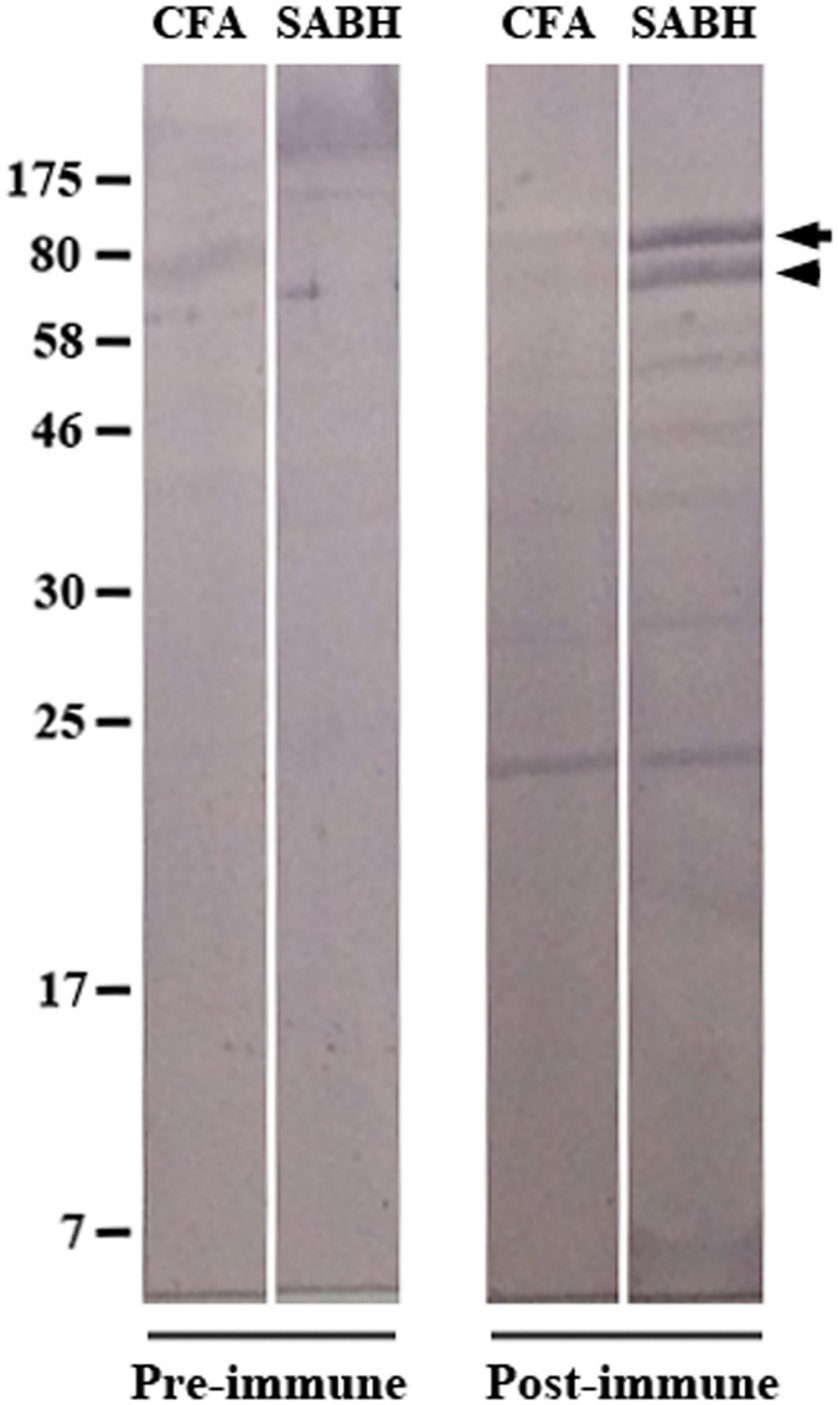
Western blot analysis of *P*. *insidiosum*’s crude protein extracts using rabbit anti- Exo1 peptide serum. Crude proteins (SABH and CFA) extracted from *P*. *insidiosum* were separated in a SDS-PAGE gel, transferred to a Western blot membrane, and probed with the rabbit pre-immune or post-immune serum. The arrow and arrow head indicate the 82- and 78-kDa band, respectively. Protein molecular weight markers (7–175) are shown in kDa. (SDS-PAGE, sodium dodecyl sulfate polyacrylamide gel electrophoresis; CFA, culture filtrate antigen; SABH, soluble antigen from broken hyphae; Pre-immune, rabbit pre-immune serum; Post-immune, rabbit anti-Exo1 peptide serum).

**Fig 7:**
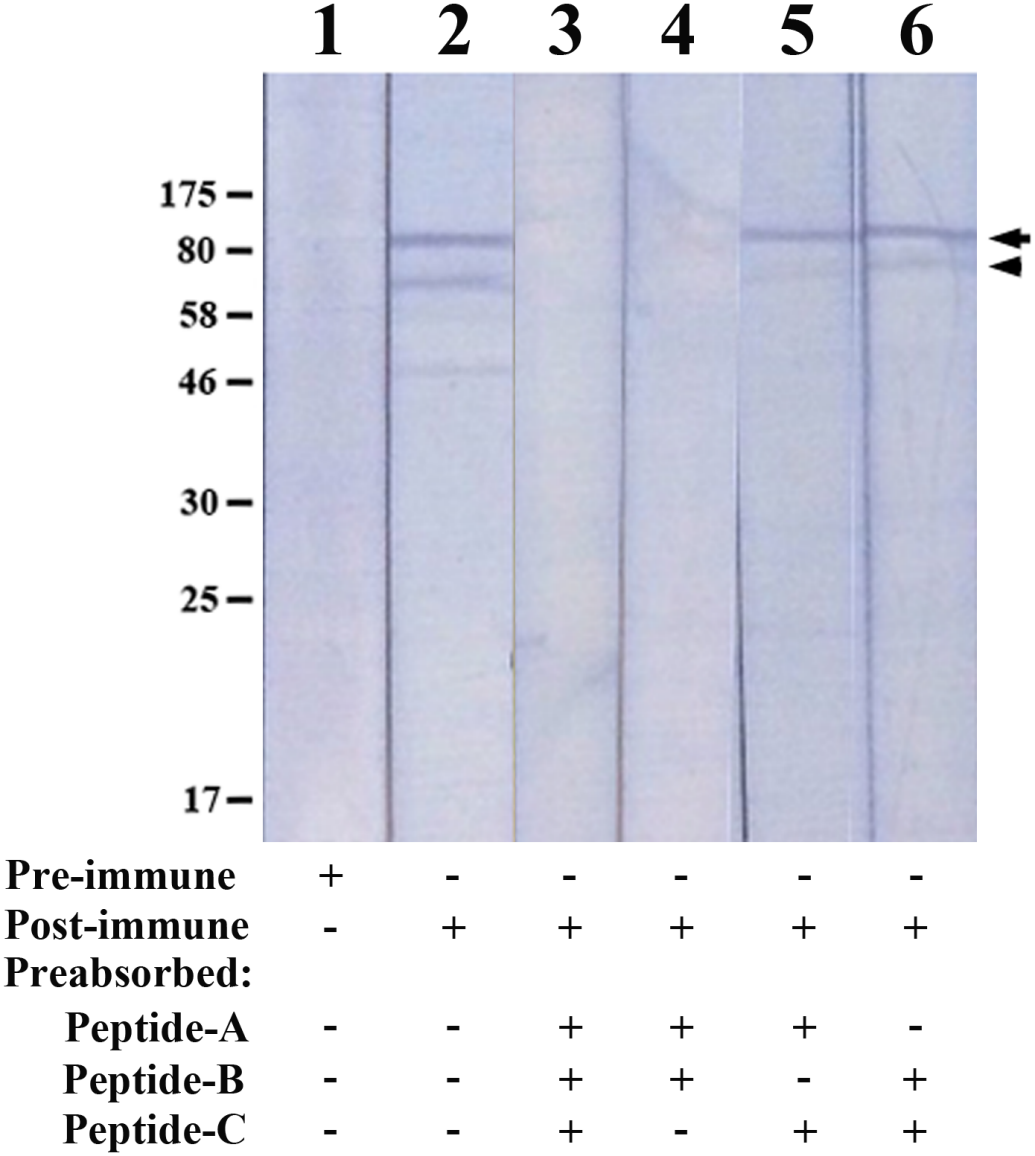
Peptide pre-absorption of the rabbit anti-Exo1 sera for Western blot analysis of *P*. *insidiosum* crude protein extracts. Separated crude proteins (SABH) of *P*. *insidiosum* were probed with rabbit pre-immune serum (Lane 1), rabbit post-immune serum (Lane 2), and rabbit post-immune serum pre-absorbed with Peptide-A, -B, and -C (Lane 3), Peptide-A and -B (Lane 4), Peptide-A and -C (Lane 5), and Peptide-B and -C (Lane 6). The arrow and arrow head indicate the 82- and 78-kDa band, respectively. [Abbreviations: SABH, soluble antigen from broken hyphae; Pre-immune, rabbit pre-immune serum; Post-immune, rabbit anti-Exo1 peptide serum; ‘+’, used as probe (pre- and post-immune sera) or used for pre-absorption (Peptide-A, -B, or -C); ‘-’, not used as probe nor used for pre-absorption]

Rabbit anti-Exo1 peptide serum was raised against the combination of Peptides-A, -B, and -C (Fig 1G). Based on ELISAs, the rabbit pre-immune serum (dilution 1:1,000) had only trace reactivity against the peptides, while the rabbit anti-Exo1 peptide serum (dilution 1:1,000) reacted strongly with each peptide (Fig 5). When the rabbit anti-Exo1 peptide serum was tested at a higher dilution (i.e., 1:64,000), Peptide-A showed the strongest immunoreactivity (Fig 5). The rabbit anti-Exo1 peptide serum was used to detect the cellular location of Exo1. The proteins in SABH (representing intracellular proteins) and CFA (representing secreted proteins) were separated by SDS-PAGE, blotted on a Western blot membrane, and probed with the rabbit anti-Exo1 peptide serum (Fig 6). The 82- and 78-kDa bands (estimated by in-gel protein markers) in SABH reacted strongly to the rabbit anti-Exo1 peptide serum, whereas the corresponding bands were faint in CFA (Fig 6), indicating that Exo1 is an intracellular protein. The rabbit anti-Exo1 serum, pre-absorbed with a combination of all peptides or Peptide-A and -B, failed to detect 82- and 78-kDa bands in SABH (Fig 7).

**Fig 8:**
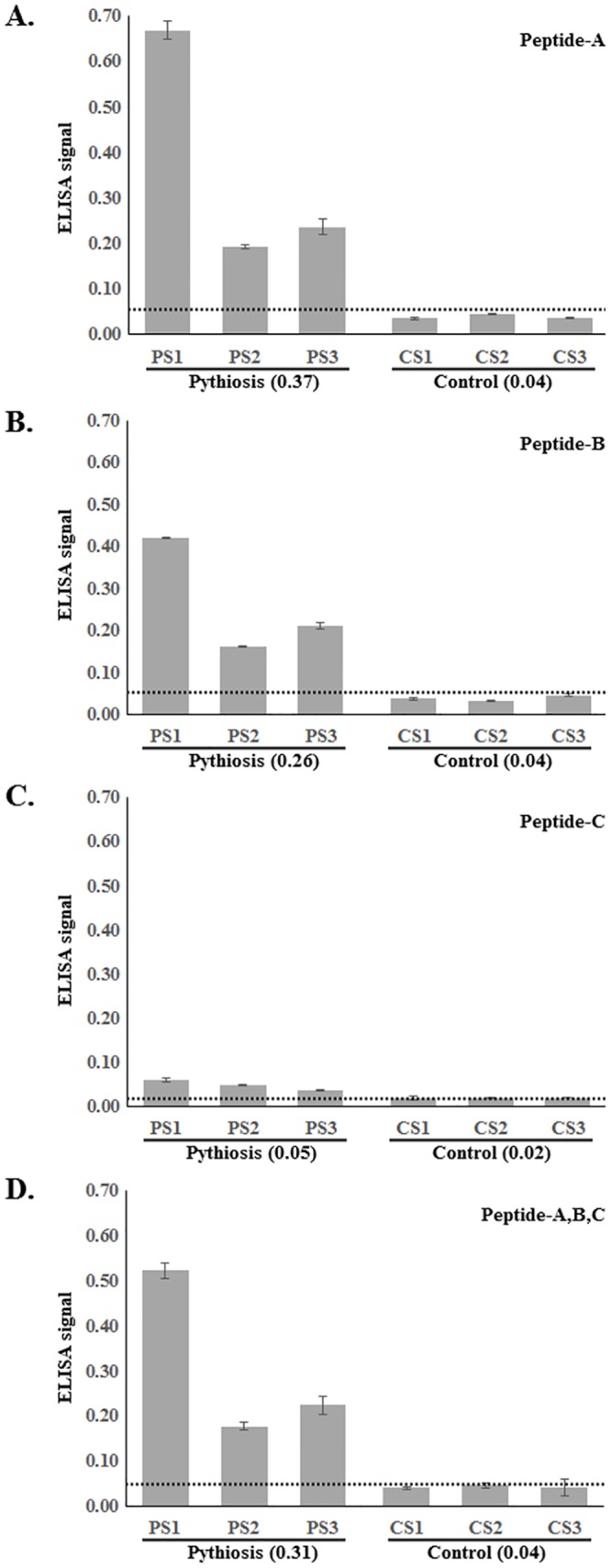
Immunoreactivity of Exo1 peptides against pythiosis patient sera by ELISA. ELISA results of serum samples from pythiosis patients (n = 3; PS1-3) and healthy blood donors (n = 3; CS1-3; control) and (**A**) Peptide-A, (**B**) Peptide-B, (**C**) Peptide-C, and (**D**) a mixture of the peptides (used to coat an ELISA plate). Number in the parenthesis is the mean ELISA signal.

Peptide-A, -B, -C, or combination of these peptides was used to coat wells of an ELISA plate, and incubated with serum samples from pythiosis patients (n = 3; PS1-3) and healthy blood donors (n = 3; CS1-3). With an exception of the Peptide-C (Fig 8C), all pythiosis patient sera reacted strongly to the Peptides-A, B, or combination of all peptides (Fig 8A, 8B and 8D, respectively), as indicated by ELSIA signals well above the cutoff. In contrast, all control sera reacted poorly to all peptides (Fig 8A–8D), as indicated by ELISA signals below the cutoff. These results demonstrate that, during *P*. *insidiosum* infection, humans mount an antibody response to peptides of the Exo1 protein.

### Exo1 exhibits glycoside hydrolase activity

**Fig 9:**
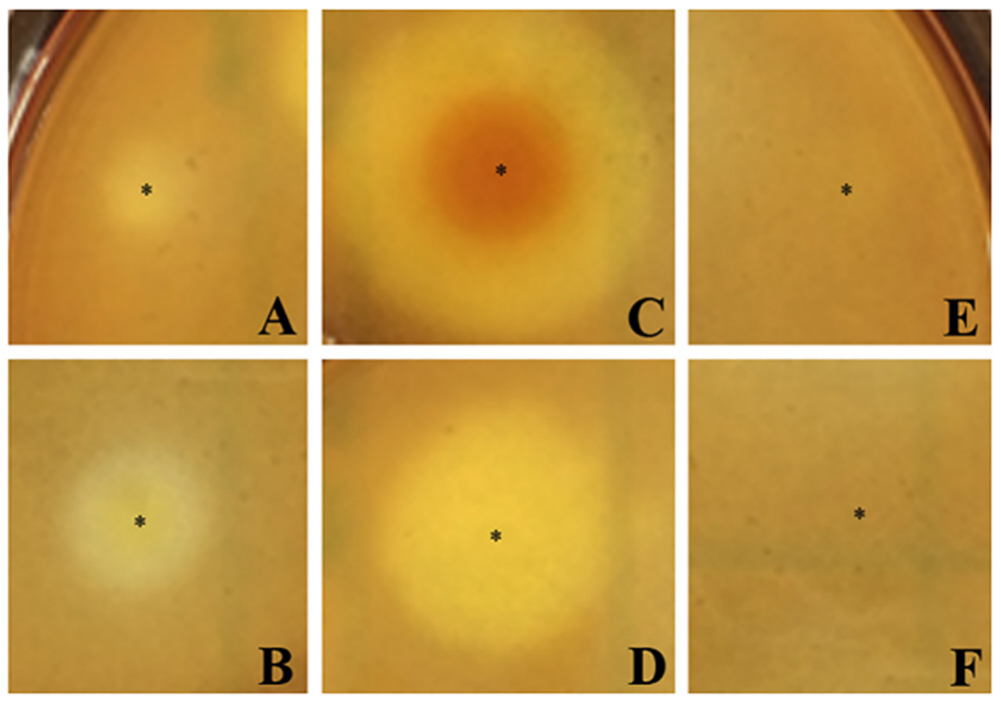
Measurement of hydrolytic activity of Exo1 by agar plate enzymatic assay: The pPinsEXO1-harboring *E*. *coli* cell suspension [1 x 10^7^ (**A**) and 1 x 10^9^ (**B**) cells/ml] and the positive controls [*T*. *harzianum*’s lysing enzyme (100 mg/ml; **C**) and *T*. *reesei*’s cellulase (100 mg/ml; **D**)] gave hydrolytic/clear zones in LB agar supplemented with laminarin. The negative controls [bacteria with the pRSET-C empty plasmid (1 x 10^9^ cells/ml; **E**) and plain LB broth (**F**)] did not produce a hydrolytic/clear zone in the laminarin plate. * indicates location where the material (bacteria, enzyme, or broth) was spotted.

**Fig 10:**
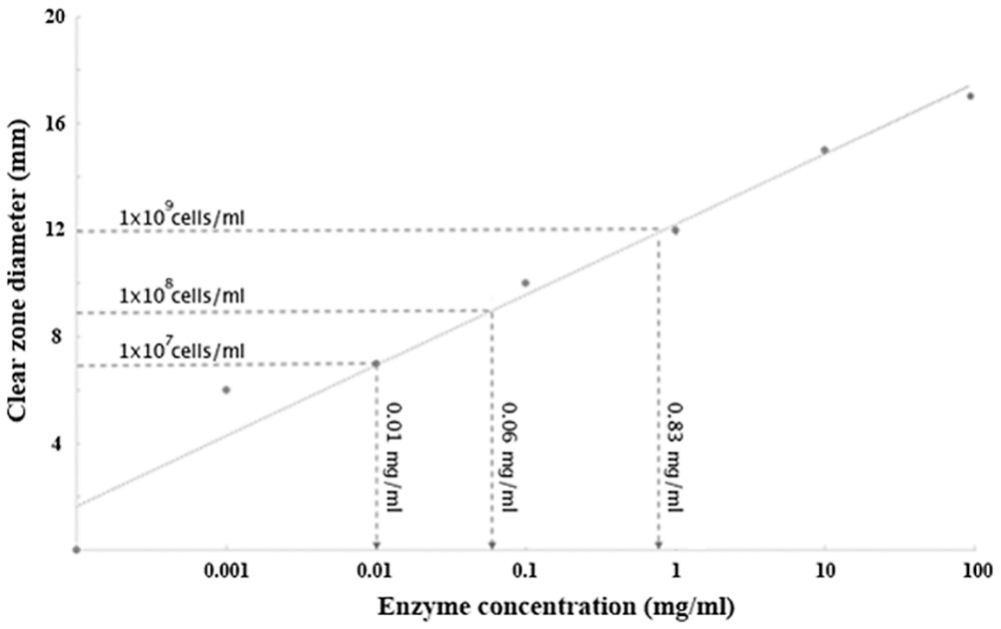
Linear correlation of clear zone diameters and amounts of hydrolytic enzyme used in the agar plate enzymatic assay. Laminarin-supplemented LB agar was hydrolyzed by various concentrations of cellulase enzyme (**X-axis**). Resulting clear zones (**Y-axis**) were visualized by staining with iodine. There is a linear relationship between the amount of enzyme and the diameter of clearing when displayed on a semi-log plot. Clear zone diameters generated by various pPinsEXO1-harboring *E*. *coli* cell suspensions (horizontal dash lines) were correlated with amounts of enzyme (vertical dash lines).

In order to demonstrate the expected hydrolytic activity of Exo1, we cloned the full-length coding region into an *E*. *coli* expression vector pRSET-C, to make pPinsEXO1, as a HIS-tagged fusion (Methods). The *E*. *coli* strain harboring pPinsEXO1 produced only trace amounts of protein (molecular weight: ~60–90 kDa) recognized by the rabbit anti-Exo1 peptide serum (S1 Fig), and nickel-immobilized affinity chromatography failed to enrich the protein (data not shown). As an alternative for demonstrating Exo1 enzyme activity, we used an agar plate assay (Methods) to measure hydrolytic activity of Exo1, being expressed in the bacteria. The pPinsEXO1-harboring *E*. *coli* (Fig 9A and 9B) and the positive controls [*T*. *harzianum*’s lysing enzyme (Fig 9C) and *T*. *reesei*’s cellulase (Fig 9D)] provided a hydrolytic/clear zone in LB agar supplemented with laminarin, in a dose-dependent manner [i.e., with higher bacterial density or higher enzyme concentration, there is a larger clearance zone]. None of the negative controls [bacteria with pRSET-C empty plasmid (Fig 9E), and plain LB broth (Fig 9F)] produced a clear zone in the laminarin plates. In Fig 10 we show a linear relationship of either cellulase or Exo1-expressing *E*. *coli* concentrations on the clear zone diameters in the laminarin plates. Similar findings were observed in the LB plate supplemented with Avicel (a microcrystalline cellulose). Taken together, these findings indicate that the transgenically-expressed Exo1 has glycoside hydrolase activity.

## Discussion

The full-length exo-1,3-β-glucanase gene of *P*. *insidiosum* (*exo1*) was cloned, expressed, and characterized. The deduced 742-amino-acid-long protein (Exo1) contains the BglC and X8 domains, suggesting a possible role in carbohydrate transport, binding, and metabolism. Results of analyzing Exo1 with the InterProScan [24] software at the Carbohydrate Active Enzymes database (http://www.cazy.org/) [38], suggests that Exo1 belongs to the Glycoside Hydrolase Family 5 (GH5), which includes enzymes with cellulase or xylanase activity [i.e., endoglucanase (Enzyme Commission number [39]: EC 3.2.1.4), xylanase (EC 3.2.1.8), exo-1,3-glucanase (EC 3.2.1.58), endo-1,6-glucanase (EC 3.2.1.75), β-mannanase (EC 3.2.1.78), and endoglycoceramidase (EC 3.2.1.123)]. GH5 enzyme-encoding genes are found in a variety of organisms (i.e., fungi and plants), but not in the human genome [40]. The agar plate enzymatic assay demonstrated that Exo1, expressed in bacteria, had the ability to hydrolyze laminarin (β-glucan; β(1→3)-glucan with β(1→6)-branches) and Avicel (high purity cellulose). These biochemical findings support the InterProScan prediction that Exo1 has a functional domain with GH5 enzyme activity (β-glucanase and cellulase).

As expected from their evolutionary histories [15], the glucanase-encoding genes from the oomycetes (including *P*. *insidiosum*) were phylogenetically grouped together and separate from the glucanase-encoding genes from fungi (Table 2; Fig 3). Among oomycetes, glucanase-based phylogenetic analysis (Fig 3) surprisingly showed that *P*. *insidiosum* was more closely-related to *Saprolegnia parasitica* (a member of the Saprolegnian lineage), than *Pythium ultimum* and also *Phytophthora* spp (*Pythium* and *Phytophthora* species are members of the Peronosporalean lineage) [41]. This paradoxical relationship may be tied to the observations that both *P*. *insidiosum* and *S*. *parasitica* are animal pathogens, while all other oomycetes included in this study are plant pathogens [42].

Changing expression of *exo1* in response to stresses, such as elevated temperature, aging, and limited carbon source, have been investigated here. The ability to grow at human body temperature (37°C) is a crucial factor for successful pathogens, including *P*. *insidiosum*. As demonstrated by quantitative real-time PCR (Fig 4), increasing the temperature, from 28°C to 37°C, significantly up-regulated *exo1* expression, suggesting that Exo1 is required as a part of pathogen’s adaptation to high temperature. In synthetic growth medium, dextrose is the main source of carbon for basic metabolisms of *P*. *insidiosum*. Aging *P*. *insidiosum* (the organism was grown in the medium for a relatively long period) up-regulated *exo1* expression (Fig 4). One hypothesis to explain an increase in Exo1 with aging or senescence is that the release of dextrose/glucose from the cell wall or intracellular β-glucan stores could support continued basal metabolism as external supplies of glucose dwindle. In contrast, growth of the organism in medium lacking dextrose from the initiation of the culture resulted in significant down-regulation of *exo1*. We propose that under the condition of no added dextrose, the metabolism of the cell is dramatically slowed, and there is likely a global effect on the expression of many genes.

Western blot analysis, using rabbit anti-Exo1 peptide serum, detected two major immunogens (~82 and ~78 kDa), and trace amounts of several lower molecular weight proteins (~20–60 kDa), in SABH (representing intracellular proteins) (Fig 6). All of these immunogens were also detected as secreted proteins of low abundance in CFA (Fig 6). These results suggests that, although small amount of the protein may be are found in the culture supernatant, Exo1 is predominantly a cytoplasmic protein. The rabbit anti-Exo1 serum, pre-absorbed with all three peptides (Peptide-A, -B, and -C), failed to detect 82- and 78-kDa proteins, whose sizes match the transcriptome-derived Exo1 homologous proteins that contains both Peptide-A and -B (UN05080 and UN00475; Table 3), in SABH (Fig 7), suggesting that the rabbit anti-Exo1 serum was specific to the proteins containing Peptide-A, -B or -C, including the 82-kDa protein, Exo1 (Table 3; Fig 2). In addition, when the rabbit anti-Exo1 serum was pre-absorbed with a combination of Peptide-A and -B (but not Peptide-A and -C, nor Peptide-B and -C), the 82- and 78-kDa proteins disappeared (Fig 7), indicating that these two proteins contain both Peptide-A and -B, which had strong antibody recognition.

BLAST searches of the Exo1 Peptide-A, -B, and -C (Fig 1G) against the 454-derived transcriptome of *P*. *insidiosum* [21] revealed that at least two of the Exo1 peptides matched two abundant transcripts (Table 3): UN05080 (number of transcript reads, 109; predicted protein size, 83 kDa) and UN00475 (17 reads; 76 kDa). Either Peptide-B or -C matched several other transcripts (Table 3): UN03240 (1 read; 33 kDa), UN01457 (1 read; 28 kDa), UN24957 (4 reads; 27 kDa), and UN22794 (1 read; 20 kDa). These transcriptome-derived proteins have high degree of sequence similarity to Exo1 (identity 88–100%; *E*-value < -87; Table 3). The two major bands on the Western blot (Fig 6) match well with the expected sizes predicted by the genes represented by UN05080 and UN00475 transcript clones (Table 3). Based on peptide mapping, sequence homology, and predicted protein size and domain, UN05080-derived protein is most similar to Exo1 (Table 3; Fig 2). According to BLAST searches against the NCBI database, the *P*. *insidiosum* transcriptome-derived protein sequences found matches to putative exo-1,3-β-glucanases from *Phytophthora* spp (identity 48–72%; *E*-value < -51; Table 2). Like other oomycetes, which have several glucanase genes in their genomes [16,17], the *P*. *insidiosum* transcriptome contains at least 6 glucanase-homologous genes that encode proteins with predicted BglC or X8 domains (Table 3; Fig 2). These homologues are expected to serve roles in carbohydrate metabolism, but their precise roles and how they may differ from each other remain to be investigated.

ELISA analysis revealed that Peptide-A and Peptide-B were strongly recognized by pythiosis patient sera (PS1-3), but not by control sera (CS1-3; Fig 8A–8B). Additionally, Peptide-A was recently reported as an efficient ELISA marker used for diagnosis of pythiosis [11]. This result confirmed that Exo1 is a major immunoreactive protein [10,12] with B-cell epitopes that trigger a host immune response during natural infection of *P*. *insidiosum*.

In conclusion, full-length exo-1,3-β-glucanase-encoding gene (*exo1*) was successfully cloned and expressed. Glucanase genes from oomycetes, including *P*. *insidiosum*, form a clade that is distantly related to the glucanase genes from fungi. Exo1 was predicted to contain the BglC and X8 domains found in proteins with a role in carbohydrate metabolism. Exo1 was characterized as a major intracellular immunoreactive protein that exhibits GH5 hydrolytic activity. *exo1* is up-regulated upon exposure to body temperature and prolonged incubation, but down-regulated upon exposure to long term carbon-depleted conditions. Exo1 contains B-cell epitopes that trigger host immune responses during *P*. *insidiosum* infections in humans. Since GH5 enzyme-encoding genes are not present in the human genome, Exo1 could be a good candidate for development of drugs or vaccines against *P*. *insidiosum*.

## Supporting Information

S1 Fig Western blot analysis of cell lysate of *exo1*-expressing *E*. *coli* and rabbit anti-Exo1 peptide serum. Cell lysate proteins prepared from the *exo1*-expressing *E*. *coli* strain, after IPTG induction, were separated in a SDS-PAGE gel, transferred to a Western blot membrane, and probed with the rabbit pre-immune (Lane: Pre-Immune) or post-immune (Lane: Post-Immune) serum. The arrowheads indicate multiple protein bands (sizes: 60–90 kDa), only present when probed with the rabbit post-immune serum.
(TIF)
