# Supplementary figures and images for: The Immunoreactive Exo-1,3-β-Glucanase from the Pathogenic Oomycete Pythium insidiosum Is Temperature Regulated and Exhibits Glycoside Hydrolase Activity
Source: PLoS One. 2015 Aug 11;10(8):e0135239. doi: 10.1371/journal.pone.0135239 (PMC4532416; doi:10.1371/journal.pone.0135239)

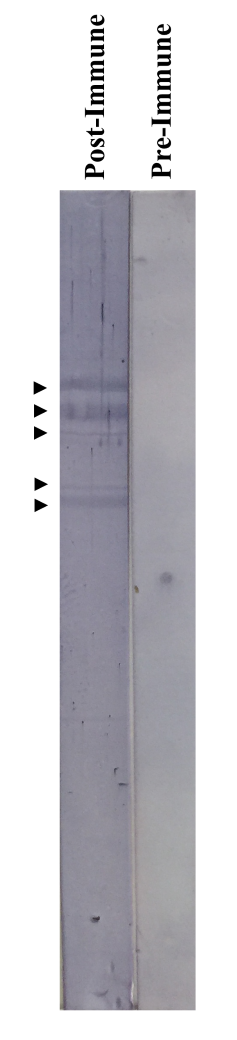

Supplement: S1 Fig — Cell lysate proteins prepared from the exo1-expressing E. coli strain, after IPTG induction, were separated in a SDS-PAGE gel, transferred to a Western blot membrane, and probed with the rabbit pre-immune (Lane: Pre-Immune) or post-immune (Lane: Post-Immune) serum. The arrowheads indicate multiple protein bands (sizes: 60–90 kDa), only present when probed with the rabbit post-immune serum. (TIF) [file pone.0135239.s001.tif]
